# Experiences of healthcare providers with eligible patients’ loss of decision-making capacity while awaiting medical assistance in dying

**DOI:** 10.1177/26323524221128839

**Published:** 2022-10-14

**Authors:** Caroline Variath, Elizabeth Peter, Lisa Cranley, Dianne Godkin

**Affiliations:** Lawrence S. Bloomberg Faculty of Nursing, University of Toronto, 155 College Street, Toronto, ON M5T 1P8, Canada.Faculty of Health and Human Services, Vancouver Island University, Nanaimo, BC, Canada; Lawrence S. Bloomberg Faculty of Nursing, University of Toronto, Toronto, ON, CanadaJoint Centre for Bioethics, University of Toronto, Toronto, ON, Canada; Lawrence S. Bloomberg Faculty of Nursing, University of Toronto, Toronto, ON, Canada; Trillium Health Partners-Mississauga Hospital, Mississauga, ON, CanadaLawrence S. Bloomberg Faculty of Nursing, University of Toronto, Toronto, ON, CanadaJoint Centre for Bioethics, University of Toronto, Toronto, ON, Canada

**Keywords:** decision-making capacity, end-of-life care, healthcare providers, medical assistance in dying, moral agency, palliative care, relational autonomy

## Abstract

**Background:**

In Canada, under Bill C-14, patients who met all eligibility requirements were prevented from accessing medical assistance in dying (MAiD) following their loss of decision-making capacity while awaiting MAiD. The changes introduced with Bill C-7 continue to limit access to patients who did not enter a waiver of final consent agreement with their healthcare providers. Little is known about the experiences with patients’ loss of capacity to consent and subsequent ineligibility for MAiD. Understanding healthcare providers’ experiences has important implications for improving end-of-life care for those with capacity-limiting conditions.

**Purpose:**

To explore Canadian healthcare providers’ experiences with end-of-life of eligible patients who became ineligible for MAiD due to their loss of decision-making capacity to consent and the relational influences on their experiences prior to the implementation of Bill C-7 in Canada.

**Method:**

A critical qualitative methodology and a feminist ethics theoretical lens guided this study. A voice-centred relational approach that allowed an in-depth exploration of how power, relationality and moral agency influenced participants’ experiences was used for data analysis. Data consisted of semi-structured interviews with 30 healthcare providers.

**Findings:**

The analysis resulted in the following four main themes and corresponding subthemes: (1) identifying factors that may result in ineligibility for MAiD due to capacity loss; (2) maintaining eligibility required to access MAiD; (3) preparing for an alternative end-of-life; (4) experiencing patients’ capacity loss.

**Discussion:**

This study highlights that while MAiD is legally available to eligible Canadians, access to MAiD and care for eligible patients who were unable to access MAiD due to their loss of decision-making varied based on the geographical locations and access to willing MAiD and end-of-life care providers. The availability of high-quality palliative care for patients throughout the MAiD process, including following the loss of capacity to consent and subsequent ineligibility, would improve the end-of-life experience for all those involved. The need to establish a systematic approach to prepare and care for patients and their families following the patients’ loss of capacity and subsequent ineligibility for MAiD is also identified.

## Background

Health Canada reported that as of January 2021, approximately 21,589 medically assisted deaths had occurred since the introduction of Bill C-14 in Canada in 2016.^1^ The legislation allowed individuals who met the eligibility criteria to request and receive medical assistance in dying (MAiD). To meet eligibility under Bill C-14, individuals were required to (1) have access to funded health care in Canada, (2) be 18 years or older, (3) have a grievous and irremediable medical condition, (4) make a voluntary request and (5) be able to provide informed consent.^2^ Safeguards included assessment of eligibility by two independent assessors, the requirement of a final confirmation of consent at the time of MAiD provision and a minimum 10-day reflective wait-period between the written request and the date of provision.^2^ The wait-period could be shortened if both assessors deemed that patients were at risk for imminent loss of capacity or death.^2^ Assessments and provisions of MAiD in Canada can only be performed by physicians and nurse practitioners.^2^ MAiD can be provided through prescription of oral medications that the patient self-ingests or through direct intravenous administration of medications.^2^ Other healthcare professionals such as registered nurses and social workers often coordinate (MAiD co-ordinators) or assist and support MAiD provisions.^3,4^

The requirement to confirm consent at the time of MAiD provision in Bill C-14 rendered many people who initially met eligibility requirements ineligible due to their loss of decision-making capacity prior to the provision of MAiD.^4^ Health Canada reported that in 2020, 34.3% of those who received MAiD had the 10-day reflection period shortened.^1^ Many practitioners, however, indicated that it was challenging to predict the risk for capacity loss.^4^ The most frequently cited reason for refusing people access to MAiD in Canada is the loss of decision-making capacity.^1,5^ Many patients, such as Audrey Parker, who publicized her fear of capacity loss and subsequent ineligibility, requested MAiD early, and others endured poor symptom management to maintain capacity.^4,6^ In response to advocacy from the public to pass ‘Audrey’s Amendment,’ and in consultation with experts and stakeholders, the Canadian Government included an amendment to waive the final confirmation of consent requirements of Bill C-14 in new legislation (Bill C-7) introduced in March 2021.^6,7^ To enact the waiver of final consent, patients must: (1) have met all legislative conditions (including reasonable foreseeability of death), (2) have been informed of their risk for losing decision-making capacity, (3) have chosen a date for the MAiD provision, (4) have provided a written advance consent to receive MAiD on the chosen date, (5) have entered into a written agreement with the providing practitioner to waive final consent following their loss of capacity and (6) have not resisted the administration of lethal medications in a meaningful way at the time or provision.^8^ The 10-day reflective wait-period safeguard was also removed.^8^

Previously, we reported the perspective of our participants on using the waiver of final consent amendment. The findings indicate that although the introduction of Bill C-7 will increase access for eligible patients, those who did not enter into a contract with their provider or those with objecting family members or healthcare providers may not receive MAiD.^4^ In addition, Bill C-7 has safeguards that prevent people with loss of decision-making capacity prior to requesting or meeting eligibility requirements from accessing MAiD, as a means to protect them from coercion.^8^ Inaccessibility to MAiD due to the loss of decisional capacity may subject patients to an end-of-life experience that does not align with their wishes for a peaceful death.

The MAiD legislation mandates that patients be given the opportunity to explore other treatment options.^2^ Health Canada reported that in 2020, 82.8% of patients who requested MAiD were already connected to and receiving palliative care.^1^ The reasons patients chose MAiD in 2020, included the loss of ability to engage in meaningful activities (84.9%), loss of ability to perform activities of daily living (81.7%) and inadequate pain control (or concern about it) (57.4%).^1,5^ People who have lost decision-making capacity after being found eligible may therefore continue to endure suffering. The Collège des Médecins du Quebec has pointed out that there are difficulties in providing appropriate end-of-life care for people who have lost decision-making capacity.^9^ Symptom management and comfort care, for example, become challenging when patients are unable to communicate verbally.^10^ The prevalence and inadequate treatment of symptoms are high among those with cognitive impairments even when receiving palliative care.^11^ Several reviews have identified that despite advancements in palliative care for patients with dementia, patients who are unable to make decisions endure poorly managed end-of-life symptom burden and suffering.^12–14^ In addition, there are disparities in access to and in the quality of end-of-life and palliative care across Canada.^15,16^ Healthcare providers often report feeling unprepared and unsupported to fulfil people’s end-of-life wishes, such as requests to die at home.^16,17^ Family members report providing care for their loved ones at the end-of-life challenging.^18^

In a report on the feasibility of using advance consents for MAiD, the Council of Canadian Academies (CCA) indicated that,

Allowing or prohibiting advance requests for MAID requires policymakers to take a position on the interplay among the concepts of autonomy (individual and relational), suffering (and the intolerability of suffering), and vulnerability (inherent and situational) created by a loss of decision-making capacity.^15^ (p. 34)

These concepts and contexts have not been explored specifically in relation to patients eligible for MAiD who have since lost decisional capacity in Canada and globally.^3^ In addition, healthcare providers may experience unique ethical and moral challenges while caring for eligible patients who later became ineligible due to the loss of decision-making capacity, as patients did not receive the end-of-life they had desired.

The purpose of this study was to explore: (1) Canadian healthcare providers’ experiences with end-of-life of eligible patients who became ineligible for MAiD due to their loss of decision-making capacity to consent and (2) the relational influences on their experiences (prior to the implementation of the waiver of final consent amendment).

## Methods

A qualitative approach using a feminist ethics lens with a focus on relationality guided this study.^4^ Relationality emerges from feminist critiques of individualistic views of autonomy and personhood.^19–21^ Focusing on relationality allowed a critical analysis of the influence of power, relationships, moral agency, and sociopolitical context on the healthcare providers’ experiences with eligible patients’ loss of capacity and subsequent ineligibility for MAiD.^19–21^ Contextual dimensions such as practice settings, policies, regulations and hierarchies on healthcare providers were also examined.

This study received approval from the Research Ethics Board at the University of Toronto (protocol no. 39865). To obtain a balanced appreciation of the relational influences on experiences, a heterogeneous sample was recruited using a maximum variation approach of purposive and snowball sampling methods.^22^ Email listservs and social media platforms of professional and voluntary organizations were used to invite participants. To take part in this study, participants were required to have had experiences with patients who were deemed eligible for MAiD and lost decision-making capacity prior to MAiD provision. Thirty participants from eight Canadian provinces who worked in various settings such as hospitals, the community, rural areas, hospice, and long-term care homes were recruited. Semi-structured interviews using a guide (see Table 1) were conducted virtually or over the telephone between November 2020 and March 2021, prior to the implementation of Bill C-7. Data analysis, using a voice-centred relational approach, consisted of two stages.^23^ The first stage involved four consecutive readings of the interview transcripts. Following an initial read for the overall plot, the researcher ‘listened’ for the concepts of power, relationality, and moral agency in the subsequent readings.^23^ The process allowed a critical analysis and documentation of each participant’s experiences. During the second stage, summarization of data and thematic analysis were conducted.^23^ Focusing on the contextual details of the heterogeneous sample yielded rich data that Malterud and colleagues^24^ refer to as ‘information power’ to explain the phenomenon being explored.^22^ A substantive appraisal approach proposed by Eakin and Mykhalovskiy, that promotes reflexive practice, highlights the importance of contextual participant characteristics and promotes the creative use of the theoretical lens, was used to enhance validity.^22^ Detailed information on participant recruitment, data collection and analysis is reported elsewhere.^4^

**Table 1.** Interview guide.

Interview guide
1. To begin, please describe your journey of becoming involved in MAiD provisions. 2. How would you describe your experiences so far with MAiD? 3. Tell me about the clinical settings in which you provide MAiD, and other members of the MAiD team, if applicable. 4. Describe a patient (without disclosing their identity) you have looked after, who was eligible for MAiD and lost decision-making capacity while awaiting MAiD. 5. Describe how you perceived the experience of the patient’s substitute decision-maker (SDM(s)) and family members when the patient was no longer eligible to receive MAiD due to loss of capacity. 6. What was it like for you and the other members of the MAiD team, if applicable, when patients had an unanticipated loss of capacity while awaiting MAiD, and as a result, became ineligible for MAiD? 7. Describe the EOL of eligible patients who did not receive MAiD due to the loss of decisional capacity. 8. How did the experiences with your patients’ inaccessibility to MAiD due to capacity loss, impact your practices and approaches?

EOL, end-of-life; MAiD, medical assistance in dying.

## Results

The participants included physicians (*n* = 13), registered nurses (RNs) (*n* = 9), nurse practitioners (NPs) (*n* = 6), and social workers (*n* = 2). Although the participants had varying clinical backgrounds (see Table 2), 17 had palliative care expertise, including acute care and community-based palliative care specialists and multidisciplinary specialist team members (*n* = 12) as well as those with palliative care as a subspecialty (*n* = 5). Five main themes were identified through analysis (see Table 3).

**Table 2.** Demographic data.

Participant (*n* = 30) characteristics		
Professional role	Nurse practitioners	6
	Physicians	13
	Registered nurses	9
	Social workers	2
Role in MAiD	MAiD assessor	2
	MAiD assessor and provider	17
	MAiD co-ordinator	6
	MAiD team member	5
Region of practice	Central Canada	12
	The Atlantic provinces	5
	The Prairie provinces	10
	The West Coast	3
Areas of practice^a^	Acute care hospital	17
	Community	18
	Long-term care home	6
	Hospice	4
	Multiple settings	8
Areas of practice (MAiD)^a^	Acute care hospital	24
	Community	24
	Long-term care home	12
	Hospice	11
	Multiple settings	23

MAiD, medical assistance in dying.

a There is some overlap as healthcare providers often practise and provide MAiD in multiple settings.

**Table 3.** Themes and subthemes.

Themes	Subthemes
Identifying factors that may result in ineligibility for MAiD due to capacity loss	• Lack of information about the prognosis and availability of MAiD. • Access-related concerns. • Constraints of the MAiD legislation.
Maintaining eligibility required to access MAiD	• Empowering patients with information. • Determining risk for capacity loss. • Balancing symptom management and risk for capacity loss. • Expediting MAiD provisions.
Preparing for an alternative end-of-life	• Discussing alternative end-of-life options. • Involving family members.
Experiencing patients’ capacity loss	• Variations in the experiences of the healthcare providers. • Challenges with patient care following their loss of capacity. • Perceived influences on family members’ experiences. • Supporting the MAiD team and family members.

### Identifying factors that may result in ineligibility for MAiD due to capacity loss

Various relational influences that delayed access to MAiD, often resulting in patients’ loss of decision-making capacity and ineligibility for MAiD, were reported.

#### Lack of information about prognosis and the availability of MAiD

Participants shared that patients often requested MAiD very late in the trajectory of their illness due to a lack of information about their prognosis and limited awareness of the option of MAiD. Many believed that the sociocultural stigma towards death made it challenging for healthcare providers to talk about death and dying: ‘I think our whole society has sanitized death or ignored the fact that we’re all going to die to the extent that people aren’t comfortable acknowledging that it’s going to happen.’ To preserve a sense of hope in patients, healthcare providers often refrained from empowering patients with information required to make informed end-of-life decisions. One MAiD provider shared:

The first person who lost capacity, my frustration was that I didn’t feel that her palliative care doctor was being honest with her. He was telling her that, you know how people will say, less than three months? Well, one day is also less than three months but so is two and a half months . . . I saw her several times, and she was clearly wasting away before my eyes.

Barriers to patients’ knowledge about MAiD such as healthcare providers’ moral objection to the intervention, perceived professional constraints or focus on prolonging life often resulted in delayed requests for MAiD. A MAiD provider shared:

The family were pretty upset that this guy had been going back and forth to the cancer clinic for a year and half, no one had brought up MAiD. He’d been talking with this family doctor for years, no one had brought up MAiD.

Healthcare providers whose personal or professional values and beliefs did not align with MAiD were believed to withhold information about its availability: ‘I think there is a lot of things where we’re allowing our personal values and beliefs to interfere in a patient’s and family’s right to know about MAiD.’ Similarly, some healthcare providers reportedly lack knowledge and training about MAiD, and therefore, were not comfortable talking about it, while others believed that discussing MAiD equated to giving up hope on the patient. In some provinces, participants expressed frustration about the stance that some professional regulatory bodies have taken about discussing MAiD:

I think one of the things that distresses me the most about this whole MAiD thing has been the attitude of [some regulatory bodies] in their interpretation of what is counselling. . . . I see my NP, nursing and social worker colleagues struggle every day with not being able to raise the topic or sometimes they don’t feel that they can even answer questions appropriately.

In addition, many health practitioners were believed to be concerned about the legal repercussions of discussing MAiD due to the clause in the Criminal Code that it is illegal to counsel someone to choose MAiD.

#### Access-related concerns

Variations were noted in the experiences with mitigating risk for capacity-related ineligibility for MAiD across the country due to disparities in access to MAiD. For instance, healthcare providers who served in remote or sparsely resourced areas were burdened with the responsibility of identifying and addressing the risk for capacity loss and ineligibility for MAiD. As a MAiD care co-ordinator shared:

The injustices of the way that the people up north are treated is something I’m quite upset about. This gentleman had requested to see our team and we saw him virtually and he made an urgent request for us to come and give him MAiD. But we had to fly there so it’s not like we could just go.

Patients who had access to well-supported centralized, multidisciplinary MAiD teams reportedly were less at risk for ineligibility due to delays and subsequent capacity loss. In addition, as some patients prefer not to have MAiD in their homes, participants often found it challenging to find locations to conduct the provisions, which became more pronounced over the pandemic. A MAiD provider stated:

My first patient was here in this clinic and many, many since, because they don’t have a reasonable place. They can’t or don’t want to be at home and they aren’t in a facility that allows it, so I offer them my clinic.

Participants expressed frustrations when their agency to provide MAiD was limited by health care institutions that did not allow MAiD provisions on their premises. One participant shared:

The fact that healthcare institutions have more rights than the patients that are housed within their walls is a big issue that we are constantly dealing with. A palliative unit is housed within a Catholic facility that is abstaining. So, the people who are the most ill, suffering with the most difficult to control symptoms, have MAiD essentially taken away from them because they’re too ill to be able to transfer out . . . that I find is disheartening on a regular basis.

Nonobjecting hospital care teams also faced resistance from healthcare team members or leaders who are conscientious objectors, often resulting in delays. A MAiD provider from an urban acute care setting stated: ‘The ward is a disaster this way, the palliative care unit. But there’s a couple other wards in the hospital here . . . we know, oh, this is going to be fun. So, it’s smoothing feathers, making sure everybody knows what’s happening.’ Similarly, some palliative care practitioners reportedly voiced their objection by refusing to bring up MAiD, make referrals to MAiD providers or perform eligibility assessments. One participant commented on the increase in the provision of services such as palliative sedation to avoid patients from choosing MAiD: ‘There suddenly became this push in the palliative care unit to give everybody palliative sedation as if somehow that would forestall MAiD.’

In addition, objection from family members reportedly delayed the MAiD process. Participants shared experiences of family members imposing their values and beliefs on patients to prevent them from accessing MAiD. Extensive meetings and mitigation strategies were required to help families accept patients’ requests. Some participants indicated that they had to remind objecting family members that the patient is not dying because of MAiD: ‘Some who have conscientious objection say, you’re not supposed to have MAiD, you’re supposed to have just the occurrence of natural death. The response to that is, they’re not actually dying of MAiD, they’re dying of advanced disease.’

#### Constraints of the MAiD legislation

Participants also found it frustrating when they could not facilitate a death that aligned with a patient’s values and beliefs due to their loss of capacity and inability to confirm consent at the time of MAiD provision as required by Bill C-14. The 10-day wait-period requirement of Bill C-14 was believed to increase patients’ chances of capacity loss and suffering while awaiting MAiD. As a provider shared:

Many people don’t get around to asking about MAiD until after they’re fed up. By the time they ask that question, and the primary care provider asks me, and I get there, they’re beyond the end of their rope and I have to talk them into waiting essentially twelve days, right? That has been frustrating, and I’ve got to be the bad guy . . . I have to turn people down.

Most participants were in support of the proposed change in the legislation to waive the final consent for MAiD, thereby minimizing ineligibility due to capacity loss for eligible patients. A few participants also believed MAiD should be available using advance requests for patients diagnosed with dementia or other neurodegenerative diseases.

### Maintaining eligibility required to access MAiD

Healthcare providers used various strategies, influenced by their clinical backgrounds and the resources available to them, to ensure that patients had timely access to and maintained capacity for MAiD. Most participants developed and implemented these strategies due to distressing experiences with patients’ loss of capacity. As implementing such strategies, many participants reported that there were less cases of capacity loss and ineligibility for MAiD.

#### Empowering patients with information

In order to minimize the delayed requests and risk for ineligibility for MAiD due to capacity loss, participants strongly advocated for patients and their families to be empowered with information about disease progression and end-of-life options earlier in the patient’s illness trajectory. A MAiD provider shared: ‘The first step in empowering patients with choices at the end-of-life requires acknowledging and talking about death and prognosis’. Even when patients were aware of MAiD, some participants acknowledged that they may not bring it up due to the perceived power and imbalances between the healthcare team and the patient, as one explained: ‘Many patients, they’re not going to raise something a doctor doesn’t talk about. They really walk into the room feeling, well, if the doctor hasn’t talked about it, maybe I shouldn’t talk about it.’ Those with a palliative care background were reportedly comfortable in helping patients make informed end-of-life care decisions. Participants who were the patients’ primary care provider often routinely had conversations about prognosis and end-of-life care, which made the process easier:

I did a MAiD case yesterday and I was [the patient’s] palliative care doctor and it was like a really lovely experience because we had a relationship. It certainly made the assessments smooth. Things can feel a bit more formal when I approach the patient just as a MAiD physician.

Many commented on the importance of being aware of the patients’ values and wishes to help them make the best possible end-of-life care decision.

Participants who were members of multidisciplinary MAiD teams believed that a team approach was ideal to assess and address the holistic needs of patients and their families about end-of-life care. Some teams had established systematic approaches to provide patients with information required to prepare for their end-of-life. Similarly, patients who were already being seen by palliative care had the advantage of being informed about and trialling palliative care, as a MAiD provider explained:

If I had to assess someone who had horrible symptom control and no access to palliative care my job really changes then, right? Like, then I would see my job as more of having to do the palliative care and if that’s not acceptable to the patient, then considering MAiD.

Many shared challenges they experienced in providing MAiD to patients who were admitted to acute care settings in which gaps in palliative and end-of-life care existed. The benefits of the MAiD legislation in enabling patients to make informed decisions about their end-of-life care were also discussed. A MAiD provider called it a positive ‘side effect’ of MAiD:

The existence of the law has allowed people who don’t get listened to, to be listened to and that in itself, even if they don’t pursue MAiD, makes them feel that they’ve got some autonomy in their life . . . it’s an interesting side effect [of MAiD].

#### Determining risk for capacity loss

Although the law allows expediting MAiD, participants indicated that determining the risk for capacity loss was often challenging, time-consuming, and resource intensive. A hospice care practitioner shared:

Our people are so fragile and it’s hard to even know sometimes what tips the balance and all of a sudden they can’t make that decision anymore . . . we see a lot of people with brain cancers that lose capacity quickly. And that’s hard.

Participants who were familiar with the patient requesting MAiD had a better sense of their risk for capacity loss. Some used indicators such as declining Palliative Performance Scale (PPS) scores and increased requirement of symptom management medications to determine risk for capacity loss. A palliative care practitioner indicated: ‘One of the major criteria that I use to say somebody probably needs to be expedited [is] if they’re starting to use so much medication that they’re at risk of losing their ability to communicate or to be lucid.’ Often, determining the risk for capacity loss required multiple visits with the patients, which were described to be challenging due to geographical and resource-related constraints. A palliative care provider explained:

If I’ve got a sick person, I’m getting over there to see them every single day to make sure that they’re not changing. So, it’s time and labour intense and it’s not planned . . . this is on top of a full-time job.

In some instances, family members and patients were encouraged to watch for signs of capacity loss, as a co-ordinator explained: ‘Part of our triage process is we say, if you notice that mum is forgetting to X, Y, and Z more often than not, you might want to give us a call.’ Others depended on patients’ primary care providers or other members of the team for information about the risk for capacity loss. Delays in MAiD provision were often caused by disagreements within the team about patients’ capacity status. Some participants solicited the help of psychiatrists to determine capacity when disagreements occurred. Determining the capacity of patients who are unable to communicate verbally was reportedly challenging, requiring resources for alternative means to communicate. A MAiD co-ordinator shared: ‘In some cases, we’ve had speech language pathology because we had a patient, in the middle of the MAiD process, had an acute stroke where they were unable to speak.’

#### Balancing symptom management and risk for capacity loss

Participants reflected on the challenges they often encountered managing patients’ symptoms while ensuring that they maintained their decision-making capacity. The quality of life of patients, while they awaited MAiD, was often diminished due to the lack of resources or misconceptions about supportive care. Participants educated patients who refused or accepted subtherapeutic symptom management medications for fear of losing capacity, about the medications and the importance of being comfortable:

I always encourage them to accept the symptom management because it’s not likely that good pain management is going to cause loss of capacity. I’ve said, sometimes the reverse is true. If you’re distressed by pain that’s not being met you’re more likely to be distracted or have other things going on that make you appear to not have capacity or to absolutely not have it.

Some discussed having to educate healthcare team members who reportedly withheld pain medications to ensure that the patient is able to provide consent. As a MAiD co-ordinator shared: ‘We’ve had cases where the nursing team has stopped their long-acting pain meds and to me that’s mind-blowing, . . . that’s not affecting their capacity at all. So now, as part of my education sessions I always mention that.’ Other strategies to ensure symptom management included staggering or changing medications and alternate doses. Some practitioners described the paradox of reversing the impact of medications or providing treatments such as fluids or steroids so that patients could regain decision-making capacity:

Sometimes as a practitioner, we kind of run into this paradox that somebody becomes too unwell to have MAiD, so we have to treat them so that they’re well enough in order to die the way they want. It’s a paradox but, you know, that’s the way it goes.

#### Expediting MAiD provisions

If patients were at risk for capacity loss, participants expedited MAiD provisions to prevent ineligibility for MAiD. Many, however, described the moral dilemmas they experienced while expediting MAiD. Relational influences on decisions to expedite included the patients’ previously expressed values and wishes, the proximity to their end-of-life, and other available symptom management strategies. Many participants indicated that they expedited MAiD often. Some, however, expressed anxiety of being judged by the other care team members when they requested an assessment for risk of capacity loss in order to expedite:

You think the patient’s ability to consent is going to expire. Then get your second assessor to reassess and agree that it should be done sooner but, that’s an awkward conversation because I just feel, you know, is the person on the other end of the line thinking, yeah, he’s just [got] a golf time two days from now and he’s just wanting to get this over with, and so I really feel like that’s anxiety provoking.

Others shared that it was important to find a balance between counselling to expedite MAiD and preventing capacity loss:

It’s those patients where I’m watching them change . . . they haven’t said no I don’t want MAiD, they [say] ‘we’ll talk about that later,’ where I’m like, if you want this to be an option we need . . . and that fine line between counselling but knowing that they’re losing the ability to do this. I find those really hard and have to come back a lot to.

Emergent provisions occurred when patients were certain of their desire for MAiD, and the patients’ death or capacity loss (or both) were imminent. Such provisions required the orchestration of various pieces: ‘We think about how quickly we can get the meds, a private room, a MAiD provider and is there anything that we can just [do to] support the patient during that time’. Some participants expressed frustration that patients and families often lost sight of a dignified death when they franticly tried to expedite MAiD. As a care co-ordinator shared:

What also has been kind of morally or ethically distressing is when they want assisted dying, their goal is for a peaceful death and they’re declining rapidly, and we can’t get the MAiD team in and the family is pushing to keep the patient alive so that they can have MAiD. I find that so bizarre.

Providing MAiD in haste reportedly compromised the opportunity to conduct extensive assessments, plan for funerals, and adequately prepare and support family members.

### Preparing for an alternative end-of-life

#### Discussing alternate end-of-life options

When participants were asked if they routinely talked about alternative end-of-life options in case MAiD was not possible, a few revealed that they had not thought about having such conversations: ‘I’ve actually never thought to have that conversation with someone. Maybe it’s something I should do’. Some practitioners assumed that the patients’ primary care teams, especially of those admitted to palliative or hospice care units, provided end-of-life care information alternate to MAiD. For participants with a palliative care background, conversations about MAiD, advance care planning and end-of-life options other than MAiD reportedly occurred seamlessly. One palliative care MAiD provider shared:

We do take time to discuss how those two deaths might look like and then if MAiD is no longer an option, this is what, family can do, this is who we would follow-up with, this is what palliative care would do.

For others, conversations about alterative end-of-life options evolved as they experienced their patients’ loss of capacity and ineligibility for MAiD. As part of the MAiD consultation, participants often inquired about the patients’ advance care plans and substitute decision-makers. Many pointed out that advance care plans and identification of substitute decision-makers are not routinely done, even when patients were receiving palliative care or were admitted to long-term care homes.

As an alternative option, some providers reportedly suggested palliative sedation. They, however, received pushback from palliative care practitioners, as patients often did not meet the strict criteria for palliative sedation. As one provider explained:

We used to bring up palliative sedation often as an option to assisted dying. We stopped doing that for a couple of reasons: one, it’s not for us to offer that, and palliative care providers were getting annoyed with us saying, ‘you brought up palliative sedation but they don’t actually meet criteria for palliative sedation and that’s for us to determine’.

Some providers acknowledged that palliative sedation may not be what the patient wants as it does not give them the sense of control they desire: ‘I think they want that kind of control, so they’re not as interested in terminal sedation, although, I think it’s important that they know that there are other options if they do lose capacity’.

#### Involving family members

Participants who engaged in alternative end-of-life care planning highlighted the importance of involving family members when possible. The loss of capacity of their loved ones reportedly caused stress for family members as they often lacked information about care following loss of capacity and were burdened with having to make decisions on behalf of patients. Often family members were unaware of patients’ values and wishes. Some participants indicated that patients and family members believed palliative care and MAiD could not be offered simultaneously, or that palliative care was not available to patients who chose MAiD. A MAiD co-ordinator shared:

The family was losing their minds, and were so upset [about the patient’s loss of capacity]. They were on an oncology floor with good palliative care. A lot of people, even healthcare staff, think that it’s either palliative care or MAiD.

Being aware of the options reportedly reassured patients and their family members that a good death was possible in the absence of MAiD. It also ensured that the families were aware of the patients’ values and wishes and reduced their burden of making healthcare decisions. Some considered failing to inform family members about death and dying as failing to support the family members’ needs:

We’ve failed the families if they’re not feeling like, okay, the goal is a peaceful death, we can do this without MAiD. The families are so distressed . . . it’s the opposite of a peaceful death and I just feel like the healthcare team as a whole is failing the families there.

### Experiencing patients’ capacity loss and ineligibility for MAiD

The experiences of family members and health care providers with a patient’s loss of capacity depended on relational factors such as the patient’s age, extent of suffering following their loss of capacity, access to information and palliative care services as well as the length and type of capacity loss the patient experienced. An RN explained:

It’s the person at home with no supports, they are dying a terrible death and they’re going to potentially linger . . . those ones to me are really difficult . . . if they are taken care of, their symptoms appear managed, the family is okay, those are the things that make it feel a little bit better.

#### Variations in the experiences of the healthcare providers

Participants indicated that most patients who had set a date were sure of their MAiD requests and did not want to endure an end-of-life in which they would lose their ability to reason, were dependent on others, and were not able to recognize family. Some participants were devastated that the patients were unable to receive an end-of-life they had envisioned, or that their existential concerns were not addressed, regardless of their perceived level of comfort or how quickly they died following their loss of capacity. A MAiD provider shared: ‘I don’t worry that if they don’t get MAiD some . . . some horrible death is going to befall them. It’s more, if they don’t get MAiD, the death that they value is not going to happen.’ Feelings of guilt and failure were pronounced when patients were at risk of enduring prolonged suffering following capacity loss: ‘In situations where patients [who] have illnesses like glioblastoma are potentially going to lose capacity and exist for weeks, no longer themselves, that’s the most emotionally wrenching cases.’ Watching family members’ pain and suffering was also challenging for some participants. As one shared:

I have trouble even thinking back to that time without starting to cry again. It was a shattering, shattering thing to be a part of. I am content I did my part, but I still feel a responsibility myself. I’ve left [the family] with complicated grief.

It was easier for some participants to come to terms with patients’ loss of capacity when the patients were perceived to be comfortable. Many believed patients were no longer suffering if they were in a comatose state, or if they were not showing signs of pain and suffering. Some found comfort in believing a dignified death was possible in the absence of MAiD or that a natural death may be more beneficial for the family members’ grieving process.

#### Challenges with patient care following their loss of capacity

Most participants who were involved in the patients’ care following their loss of capacity believed that they were able to successfully manage pain and symptoms. Patients who had seamless access to MAiD and palliative care reportedly had better symptom management. The ineffectiveness of palliative care and palliative sedation for some patients was discussed, however. A palliative care provider explained:

In some instances people suffer a lot and families suffer a lot and for days and days they’re just sitting there waiting for the patient to die and you know, we treat their symptoms as best we can, but sometimes we still can’t alleviate their suffering completely.

In addition, patients in some settings often lacked access to end-of-life care following their loss of capacity as a palliative care RN shared:

Unfortunately, he didn’t die for a week, he was on palliative care, right? They hydrated him. The healthcare facility he went to was not that experienced with palliative care. I know that food and water actually hurts the body when you’re dying. I’m not sure if the staff there knew so much.

Participants who were MAiD consultants were often unaware of the incapacitated patients’ end-of-life experience and assumed that their needs were looked after by other care teams. The importance of arranging support or transfers to appropriate care facilities for patients who were not connected to palliative care following their loss of capacity to consent was discussed: ‘What we did right away was, we were in contact with the local homecare and spoke to them about palliative sedation and supporting those final end-of-life care moments and so their team stepped right up.’ When possible, those who had palliative care backgrounds or were the patients’ primary care providers continued to provide end-of-life care following patients’ loss of capacity. One provider discussed the challenges they faced ensuring good palliative care for their patients from palliative care practitioners who conscientiously objected to MAiD:

Someone did lose capacity and the palliative care doctor was a conscientious objector and would basically hang up the phone on the nurse if I was there. The family were very aware of this distress between the two of us. So, it was a very unpleasant death.

Participants, however, in this study who had palliative care backgrounds considered MAiD an extension of their role:

A duty and a responsibility as a healthcare provider in both palliative and MAiD situations to give people access to relief. Now in palliative care you give them relief until they die and with MAiD you give them relief with death.

#### Perceived influences on family members’ experiences

Participants reported that family members who supported MAiD and were aware of the patients’ values and wishes found it challenging to accept patients’ ineligibility for MAiD. Some, especially those who had struggled to come to terms with MAiD, expressed frustration and anger towards the healthcare team. One provider shared:

He actually started screaming and threw things down the hallway . . . he was completely out of control. He kept talking about how all this time he worked to get his head around it, everything was done, [the patient] always wanted this, and why it couldn’t it happen, and this is dreadful.

Some family members reportedly felt accountable for not being able to fulfil patients’ wishes by identifying and reporting early signs of capacity loss. One participant explained:

They feel a lot of accountability and say, ‘I guess we should have called you yesterday. I don’t know why we waited.’ I think it’s a burden for them to bear because they want to honour and respect their loved one’s wishes and maybe they have the sense that they failed them.

Family members who were informed of patients’ risk of capacity loss and alternative end-of-life options, and those who received support from the healthcare team were more accepting of the natural death. Those opposed to MAiD for religious or personal reasons reportedly felt relief as they were more comfortable with a natural death or because it provided certainty that the death was not premature.

In the absence of adequate support, participants shared that family members were burdened with managing symptoms. In a few instances, families desperately wanted MAiD to be provided due to caregiver exhaustion. Sitting vigil during the protracted deaths of patients was described to be challenging for family members, especially if patients appeared to be suffering. Participants indicated that family members may experience helplessness as basic acts of care and comfort such as providing food, water, and touch are not likely to increase patients’ well-being at the end-of-life. Family members were described to be at risk for complicated grief due to their inability to say their final goodbyes, especially when patients were incoherent or aggressive following their loss of capacity. A social worker shared:

If someone doesn’t have capacity, then we as providers decide that we can’t give them MAiD, what is the obligation for the continuation of care to that patient and family to make sure that person has a peaceful and dignified death? I think that is a critical question MAiD programs have to ask, right? What is our role and obligation to these folks?

#### Supporting the MAiD team and the families

An interdisciplinary team approach was considered the most effective to support patients and families, as well as the team members throughout the MAiD process and following patients’ capacity loss and ineligibility. A participant shared:

Yeah, so we’re lucky enough to do everything as part of a multidisciplinary team and so we have a doctor, a nurse and a social worker . . . we come as a team. We can support each other as well as the family and that sort of has been a really good approach for us.

Participants who worked with palliative care providers who support the availability of MAiD experienced a better working environment than those who did not. Participants felt that having their colleagues to debrief and share challenging experiences with such as the patients’ loss of capacity was helpful. Healthcare providers who worked alone in communities, especially in the early days, lacked support and often felt isolated in their role. Practitioners who cared for MAiD patients at the bedside while they were in the process of receiving MAiD, but were not part of the MAiD team, indicated that they often felt unsupported by the MAiD team. Some institutional care teams that depended on external MAiD teams developed their own coping strategies: ‘We provide support to the staff because this experience is emotional on our team. So, we provide help with the flow of the floor and give the staff some breathing room to do a debrief.’

In terms of supporting family members, some care teams indicated that it was not part of their job description or that they did not have resources to follow-up with family members following MAiD provisions or patients’ ineligibility for MAiD. A hospice RN, whose patients were looked after by an external MAiD team, indicated: ‘This other team had come in, done their thing and left. We were left with the families to console them, but we weren’t really part of the [MAiD] process, so, it was very disconnected and disjointed.’ Some participants ensured that they comforted the family members by checking in on them, and by providing referrals to grief support systems. As an NP shared: ‘Either they can experience a traumatic event without me being sensitive to their needs or they can be guided through a traumatic event with my help. I found spiritual care invaluable.’ A few participants indicated their teams had identified a gap in the follow-up support that family members received and were in the process of requesting additional resources to better support the family members.

## Discussion

To our knowledge, this study is the first in Canada to report the experiences and perspectives of healthcare providers with eligible patients’ loss of capacity and subsequent ineligibility for MAiD and provides important insights on various relational factors that influenced participants’ experiences and perspectives. Loss of capacity to consent and the related ineligibility for MAiD prevented patients from receiving the end-of-life experience they desired and put them and their families in a vulnerable state. Feminist theorists emphasize the importance of considering humans as relational and often vulnerable beings, whose experiences are influenced by complex relationships with others, such as the healthcare team and their family.^25^ The findings of this study reveal that patients’ access to and experiences with the MAiD process are dependent on and influenced by the legislation, professional regulations, the moral comfort level of the healthcare providers, institutional policies as well as the involvement of family members.

While reflecting on experiences with eligible patients’ loss of capacity, participants expressed concern and frustration about sociopolitical barriers and professional limitations that delayed patients’ ability to make autonomous, informed decisions and impeded access to MAiD. For instance, as reported in previous studies, end-of-life conversations and discussions about poor prognosis are still challenging for healthcare providers in general.^26,27^ Patients’ lack of knowledge about their prognosis and the availability of MAiD resulted in delays and potential ineligibility for MAiD.^26,28^ Similarly, because assessors and providers participate in MAiD on a voluntary basis, disparities in access to MAiD across Canada exist.^27,28^ Many remote and under-resourced areas do not have healthcare providers willing to facilitate MAiD provisions. In addition, the personal values of healthcare providers, institutions that did not allow MAiD on their premises, as well as perceived professional restrictions and liabilities often obstructed patients’ access.^28^ Objection from family members or the community has also delayed or obstructed MAiD requests. Such obstacles impacted patients’ ability to make autonomous decisions and healthcare providers’ agency in fulfilling patients’ wishes. Identifying and addressing these barriers will continue to improve access to MAiD under the current legislation (Bill C-7), as patients are required to have decision-making capacity to request MAiD.^8^

Beyond providing MAiD, it was important for participants to ensure that patients and families had a ‘good’ end-of-life experience. Constraints of the law and inequities such as lack of access to health care negatively impacted patients’ experiences with MAiD, however. Safeguards in the MAiD legislation exist to protect patients from misuse and coercion. They, however, may negatively impact some patients’ quality of life or access to MAiD. For instance, participants reported that MAiD safeguards, such as the 10-day wait-period and final confirmation of consent requirements in Bill C-14, caused undue stress because of the related fear of losing decision-making capacity. Patients often suffered due to the perceived impact of symptom management medications on capacity, or while being transferred out of palliative and hospice care settings as result of objecting teams or institutions. Although participants anticipated that Bill C-7 may mitigate some of these concerns, some believed it could impact patients’ opportunities to change their minds and healthcare providers’ moral agency in providing MAiD.^4^

High-quality palliative care was considered important to maintain a good quality of life while patients awaited MAiD. The stance of the Canadian Hospice Palliative Care Association, as well as the resistance from palliative specialists who are conscientious objectors may have a detrimental impact on patients, however.^28,29^ For instance, in alignment with Munro *et al.*,^30^ this study identifies the need to educate patients, family members and healthcare providers that MAiD and palliative care are not mutually exclusive. Some participants indicated that patients who have chosen MAiD and are under the care of palliative care providers who were conscientious objectors may be at risk for suboptimal care while awaiting MAiD. Importantly, many participants in this study had some palliative care background. They described their efforts to ensure symptom management using the principles of palliative care, while maintaining patients’ decision-making capacity. Contrary to palliative care being incompatible with MAiD,^31–33^ these participants believed MAiD was an extension of palliative care. In a study by Bélanger *et al.*,^33^ palliative care physicians anticipated that MAiD would impede a thorough evaluation of patients’ suffering; however, participants in this study revealed that the requirements of MAiD increased patients’ opportunities to be listened to and to have their needs addressed holistically. Similar findings were reported by others, including by Beuthin and colleagues, who referred to this care as ‘rediscovering the art of medicine.’^34,35^ Like others, palliative care providers in this study took comfort in knowing that patients who continued to suffer physically or existentially while receiving palliative care had an option to end their suffering through MAiD.^28,31^ Non-palliative care participants and those who were not patients’ primary care provider ensured that patients who were ineligible for MAiD as a consequence of capacity loss had access to palliative and supportive care. In addition, consistent with previous reports, this study revealed that some patients had not been seen a palliative care specialist or team prior to their MAiD request due to a lack of access in remote locations or knowledge about such services.^15,30^ Our findings indicate that challenges to maintaining patient comfort resulted in distress for health care providers.

Participants developed strategies to monitor for the risk for capacity loss or instructed the patient and family members to monitor and report changes. Similarly, they assessed the impact of symptom management medications and treatments, modifying them when necessary to prevent related changes in capacity. Some participants described the paradox of having to treat patients who were deteriorating in order for them to regain capacity and receive MAiD. Palliative care providers reported using PPS scores and the increasing requirement of symptom management medications as indicators for impending capacity loss. A similar finding was reported by Selby *et al.*^35^ who suggested that the PPS score is a good indicator for capacity loss, recommending close monitoring of patients with a PPS score of 40% or lower. In addition, although Bill C-7 would allow MAiD provisions using the waiver of final consent, many participants indicated that they would continue to watch for capacity loss and expedite MAiD if the patient desired. Some participants anticipated challenges with MAiD provisions in the absence of a final confirmation of consent with patients, while others believed MAiD was more meaningful when patients were able say their goodbyes to their loved ones.^4^

In 2019 and 2020, approximately 34% of patients who received MAiD had the 10-day reflection period shortened, mainly due to the potential for capacity loss.^1,5^ Participants’ encounters with expediting MAiD shed light on challenges and relational influences on healthcare providers’ and patients’ decisions to shorten the wait-period.^3,36^ Prognosticating risks for capacity loss were considered challenging as patients often lose capacity unexpectedly.^4,26^ Decisions to expedite MAiD were easier when there was an established relationship between the patient and healthcare providers, and when the patients’ values and wishes were well known. The risk for capacity loss and ineligibility and the possibility for expediting MAiD were not consistently communicated with patients and their family members, requiring healthcare providers to have difficult, sensitive conversations with patients who were at risk for capacity loss. Striking a balance between counselling for an early death and preventing ineligibility for MAiD was morally burdensome for some healthcare providers. In addition, the process required two independent re-assessments as well as the orchestration of MAiD provisions. Such strategies required extensive time and resources, which many assessors and providers lacked, as they took part in MAiD provisions outside of their regular, full-time jobs.^27,28,37,38^ Some participants in this study believed that education about the dying process and symptom management measures available to patients may minimize requests for expediting MAiD.

Healthcare providers have reported the challenges and frustrations of declining MAiD to otherwise eligible patients.^4,34^ Their experiences with patients’ loss of capacity and subsequent ineligibility were influenced by personal values, relationships and contextual factors.^19,20^ For example, those who had established relationships with the patients and family members were immensely affected, especially if the patients had endured suffering or a protracted death following their ineligibility and if it impacted the family members’ grieving process. Similarly, participants whose values aligned with that of their patients and those who believed it was important to uphold patients previously established wishes found it challenging to come to terms with their patients’ capacity loss and ineligibility for MAiD. Participants who believed patients could be kept comfortable following capacity loss or that it was only family members who suffered following the patient’s capacity loss were not impacted as much, especially if patients did not appear to be suffering. Guilt and sadness were heightened when capacity loss and ineligibility occurred due to geographical or resource-related constraints as opposed to an unexpected or sudden loss of capacity.

Participants indicated that the responsibility for end-of-life decision-making on behalf of patients who are ineligible for MAiD due to their loss of decisional capacity falls on their designated substitute decision-makers and healthcare providers. Importantly, personal values and views about life and death influence people’s end-of-life decisions.^16^ Decisions, however, made by substitute decision-makers and healthcare providers may not reflect the values or beliefs, as well as the autonomous choices of the patient. Advance care plans may alleviate some of the burden that family members and healthcare providers experience by providing direction for patient-centred decision-making;^15,16,18^ however, participants shared that advance care plans were not routinely established with patients when they had capability. According to Downar *et al.*,^16^ many physicians lack formal training and are unsure of how to complete advance care plans with their patients. Our findings indicate that making decisions on behalf of patients who have lost decision-making capacity in the absence of previously established directions from patients was believed to increase the burden and suffering that family members experience. Such directives would be valuable for families to accept MAiD provision following the patients’ loss of decision-making capacity, using the waiver of final consent amendment with Bill C-7.^4^

Many studies have reported the lack of bereavement support available to family members of patients who have chosen MAiD.^37,39^ The grief experience of family members of patients who have lost capacity and are rendered ineligible for MAiD may be complicated and unique. Some participants in this study indicated that they were not involved in the continued care of patients and their families following the patients’ loss of capacity. Only a few continued to check on the needs of the family members or offer bereavement support or counselling. Concerns about the lack of support for family members in this study were raised by MAiD co-ordinators, RNs, and social workers. As reported in other studies, family members looked after by a team experienced better support throughout the MAiD process, including following patients’ capacity loss.^27,37^

### Implications for practice

Although Bill C-7 was intended to increase access to MAiD for patients at risk for capacity loss, as reported in our previous paper, many patients may continue to be ineligible for MAiD if they did not enter into a contract prior to their loss of capacity or if they lost capacity prior to being deemed to have a reasonably foreseeable death.^4^ Other jurisdictions require some patients to have decision-making capacity at the time of provision.^15^ The findings from this study can help improve the experiences of the healthcare providers, patients and family members following the patients’ loss of capacity and ineligibility for MAiD.

Only a few participants used a systematic approach to prepare patients and their families for the possibility of capacity loss and ineligibility for MAiD by routinely discussing alternative end-of-life options or ensuring that patients had advance care plans in place. Consequently, this study highlights the need to improve end-of-life conversations and standardizing advance care planning to minimize delayed requests for MAiD and to direct care in the event that MAiD cannot be provided.^4,27^ In addition, the process of effective (timely and successful) referrals needs to be standardized and strengthened across Canada.^28^ There is a need for change to support access to MAiD for patients admitted to healthcare organizations that do not allow provisions on their premises.^28^ Similarly, professional organizations should define roles and provide guidance to healthcare providers to participate in and discuss MAiD. A standard process to prepare patients and family members for end-of-life following patients’ loss of capacity would improve the experience of everyone involved. A team approach to the MAiD process that includes access to palliative care^40,41^ is recommended to ensure a holistic approach to care, including follow-up care for patients and their families. Establishing teams and enhancing the experiences of all those involved in MAiD require adequate resources, remuneration and support. Enhancing end-of-life care for those who have lost capacity to consent also requires increased access to palliative care for under-resourced and remote areas.

### Limitations

This study was conducted prior to the introduction of Bill C-7, which is intended to decrease the number of patients ineligible for MAiD following capacity loss. The findings, however, are important for patients who do not meet the criteria to waive final consent, as well as those who continue to be ineligible for MAiD in Canada due to a lack of capacity, as well as for patients in other jurisdictions that require patients to have capacity at the time of provision. The intent of this study was to suggest ways to improve the care of patients who have lost capacity to be eligible for MAiD, as well as to identify strategies to support their family members. In order to fully support patients and their families, however, their experiences also need to be explored. It would also be important to learn about the perspectives of healthcare providers who are not involved in or have conscientious objections to MAiD.

## Conclusion

This study highlights that patients’ end-of-life decisions and experiences are influenced by various relational factors. While MAiD is legally available to eligible Canadians, access to MAiD is unevenly distributed across the country. Similarly, end-of-life care for eligible patients who were unable to access MAiD due to their loss of decision-making capacity varied based on the availability of care teams, geographical location and family support. The findings from this study highlight that the best approach to end-of-life care is to offer high-quality palliative care while patients are awaiting MAiD or following their loss of capacity for MAiD. While the introduction of Bill C-7 has improved the opportunities for eligible patients to receive MAiD, this study points out the need for policies and resources to improve knowledge about and access to MAiD and other end-of-life care options. The study also identifies the need to establish a systematic approach to prepare and care for patients and their families following the patients’ loss of capacity and subsequent ineligibility for MAiD. Advance directives for MAiD in some circumstances may help improve access to MAiD. While considering increasing access to MAiD using advance directives, it would be important to address the identified gaps in access to end-of-life options and care for patients and their families who have chosen MAiD.

## Declarations

**Ethics approval and consent to participate:** Ethics approval was obtained from the University of Toronto’s Research Ethics Board (REB) (protocol no. 39865). All participants provided informed consent.

**Consent for publication:** Not applicable.

**Availability of data or materials:** Additional quotes that support this study are available from the corresponding author (CV) on reasonable request, subject to privacy and confidentiality commitments.
